# Case Series of Fertility Treatment in HIV-Discordant Couples (Male Positive, Female Negative): The Ontario Experience

**DOI:** 10.1371/journal.pone.0024853

**Published:** 2011-09-28

**Authors:** Trent Newmeyer, Sandy N. Tecimer, Denise Jaworsky, Steven Chihrin, Kevin Gough, Anita Rachlis, James Martin, Saira Mohammed, Mona R. Loutfy

**Affiliations:** 1 Brock University, St. Catharines, Ontario, Canada; 2 Women's College Research Institute, Women's College Hospital, Toronto, Ontario, Canada; 3 University of Toronto, Toronto, Ontario, Canada; 4 University of Western Ontario, London, Ontario, Canada; 5 St. Michael's Hospital, Toronto, Ontario, Canada; 6 Sunnybrook Health Sciences Centre, Toronto, Ontario, Canada; 7 Southern Ontario Fertility Technologies, London, Ontario, Canada; Indiana University, United States of America

## Abstract

The success of combination antiretroviral therapies for the treatment of human immunodeficiency virus (HIV) has resulted in prolonged life expectancy (over 40 years from diagnosis) and an improved quality of life for people living with HIV. The risk of vertical HIV transmission during pregnancy has been reduced to less than 1%. As a result of these breakthroughs and as many of these individuals are of reproductive age, fertility issues are becoming increasingly important for this population. One population in which conception planning and reduction of horizontal HIV transmission warrants further research is HIV-discordant couples where the male partner is HIV-positive and the female partner is HIV-negative. Sperm washing is a technique carried out in a fertility clinic that separates HIV from the seminal fluid. Although sperm washing followed by intrauterine insemination significantly reduces the risk of horizontal HIV transmission, there has been limited access to the procedure in North America. Furthermore, little is known about the conception decision-making experiences of HIV-discordant couples who might benefit from sperm washing. Chart reviews and semi-structured interviews were completed with 12 HIV-discordant couples in Ontario, Canada. Couples were recruited through HIV clinics and one fertility clinic that offered sperm washing. Participants identified a number of factors that affected their decision-making around pregnancy planning. Access to sperm washing and other fertility services was an issue (cost, travel and few clinics). Participants identified a lack of information on the procedure (availability, safety). Sources of support (social networks, healthcare providers) were unevenly distributed, especially among those who did not disclose their HIV status to friends and family. Finally, the stigmatisation of HIV continues to have a negative affect on HIV-discordant couples and their intentions to conceive. Access to sperm washing and fertility service is significantly limited for this population and is accompanied with a number of challenges.

## Introduction

The progression of HIV disease has been significantly altered by the use of combination antiretroviral therapy (cART). With appropriate access to cART, HIV can be managed as a chronic disease [1], [2]. As the medical treatment of HIV improves, a holistic approach requires also addressing the social and psychological needs of people infected and affected by HIV. One of these needs, support for the desire to have children, is the subject of this article.

Historically, couples living with HIV have been met with little support and even discouragement in the pursuit of pregnancy planning options [3]. This resistance has persisted despite significant medical advances that have both increased life expectancy after HIV diagnosis at least 40 years and reduced the risk of vertical (HIV-positive mother-to-baby) transmission to <1% [4].

Studies have found that the level of desire to have children amongst people living with HIV (PLWHIV) is comparable to that of the general population [5]–[7]. In Canada, there are an estimated 66,000 PLWHIV, and the vast majority of them are of reproductive age [8]. Despite the demonstrated interest in having children amongst PLWHIV, the medical community has continued to be slow to support PLWHIV in pursuing their right to a healthy pregnancy. There is even less research and knowledge aimed to support men living with HIV who desire to have children. For example, a UK survey of men living with HIV found that only 9.4% were given medical advice on reproduction [9].

For men living with HIV who desire to father children, a key clinical issue is the prevention of transmission to their female partners. If the partner is HIV-negative, it is crucial to prevent her from becoming infected with HIV. If she is HIV-positive, co-infection with another strain of HIV should be prevented. One procedure to prevent horizontal transmission from an HIV-positive man to his female partner is sperm washing. Sperm washing can be used for couples where the man is HIV-positive and the woman is HIV-negative (hereafter, HIV-discordant couples). Sperm washing is a process that employs centrifugation and swim up techniques to separate sperm from HIV found in seminal fluid. The washed sperm is then transferred directly to the woman's uterus in a procedure called intrauterine insemination (IUI). Although there is no guarantee that the HIV can be completely eliminated from the collected semen sample using sperm washing, and theoretical transmission risk exists, a landmark European study documented no cases of HIV transmission to mother or infant in over 3,300 attempts [10]. Sperm washing for HIV-discordant couples was pioneered in Milan in 1989 [11], and there have been no documented cases HIV transmission using it to date.

Despite the negligible risk of HIV transmission to both mother and child in sperm washing, few clinics worldwide, and even fewer in North America, currently perform the procedure for HIV-discordant couples. In a survey of fertility clinics in Canada, only 14 clinics (61%) out of 23 provided specific fertility services including sperm washing for PLWHIV and only 10 (43%) offered sperm washing to discordant couples [12]. While the research on the fertility desires and expectations of PLWHIV is expanding, there is a paucity of literature on the experiences of PLWHIV who undergo fertility procedures [13]. This paper seeks to document the experiences of HIV-discordant couples in Ontario, Canada who accessed sperm washing services at fertility clinics.

### Healthcare, Fertility Desire and the HIV Community

Studies have found that healthcare providers (HCP) are generally not supportive of reproductive desires and actions of PLWHIV [14]. In South Africa, PLWHIV were unlikely to discuss their reproductive intentions with HCPs given the anticipated negative reaction [15]. In a survey of 32 men living with HIV in London, almost half (41%) felt they would experience discrimination if they had conceived a baby and 25% would likely conceal their HIV status at antenatal clinics given this perceived discrimination [9]. In contrast, in Vietnam where there is more social pressure to continue the familial lineage, HCPs were supportive of the reproductive decisions made by couples living with HIV [16]. A recent study of the attitudes of Canadian HCPs towards assisted reproductive technologies found most physicians (>80%) had a positive attitude towards pregnancy and adoption for PLWHIV [17]. Research on fertility services available to PLWHIV in Canada found that access to fertility treatment was limited and regionally dependent [12]. Multivariable analyses of a survey of PLWHIV in Canada found that male PLWHIV, LGB, and those from small urban/rural areas were less likely to: expect children in the future, know about conception services, and speak to healthcare providers about pregnancy planning [18].

While most research on HIV and fertility has focused on women, little attention has been given to the fertility desires and actions of men living with HIV. In a systematic review of HIV and fertility desires, only 7 out of 29 articles included samples of men and women and only two focussed solely on men [19]. Research demonstrates that the reproductive intentions of women living with HIV are significantly impacted by their male partners [16], [20]–[22]. A study of HIV positive men in Brazil found that 56% had children already, 13% of which were born after the man's diagnosis [14]. Studies conducted in Brazil, the US, the UK, and South Africa have reported that 43%, 28%, 44% and 36%, respectively, of men living with HIV had a desire to have children [7], [9], [14], [23]. Factors linked to increased desire included being single, younger age, fewer number of children and better self-reported health [23].

Stigma and lack of disclosure can lead PLWHIV to have children as a means to hide their HIV status [15]. This pressure is often culturally and gender specific, with an expectation in many cultures that women should have children under “normal” circumstances [15], [21]. In some cultures men have also felt similar pressures to have children [16]. The expectation to have children combined with lack of disclosure of HIV status leads some couples to attempt pregnancy via unprotected sexual relations thereby risking horizontal transmission [6], [24].

The stigma related to PLWHIV having children has been identified as a significant concern for these couples in many studies [9], [15], [16]. In one study, community attitudes were that PLWHIV should not have children, yet these attitudes were associated with not knowing any PLWHIV and an ignorance surrounding the actual risks of HIV transmission in assisted reproductive technologies and pregnancy [25].

## Methods

Operating from a phenomenological perspective, we wanted to understand the experiences of HIV-discordant couples who underwent, or expressed interest in, fertility treatments with sperm washing. We conducted chart reviews and semi-structured interviews of the study population of 14 HIV-discordant couples in Southern Ontario between 2007 and 2009. One couple withdrew from the study and one only had a chart review conducted, therefore 12 interviews and demographic data from 13 chart reviews are reported. The study received ethics approval from each associated institutional research ethics board and informed consent was obtained prior to the commencement of any research activities.

### Respondent Population and Recruitment

Inclusion criteria were couples that: 1) were at least 18 years old, 2) were HIV-discordant, where the female partner was HIV-negative and the male partner was HIV-positive, 3) were interested in conceiving, and 4) had visited a fertility clinic in Ontario regarding consideration of sperm washing as a method to reduce horizontal HIV transmission. Recruitment was conducted through one of the only two clinics that offered sperm washing in Ontario as well as HIV HCPs in Ontario. Eligible HIV-discordant couples were contacted initially by either the fertility clinic or HIV HCPs and were provided with details of the study. Participants who expressed interest and verbally consented were contacted by a research team member who provided additional study information, determined eligibility, obtained informed consent and conducted the interview. Given the sensitive nature of the study topic, discretion was required when attempting to contact potential participants [25].

### Qualitative Data Collection

Qualitative data was collected through in-depth semi-structured interviews with each couple, either in person or over the phone. Phone interviews were also offered (and accepted by three couples) to help overcome challenges of distance, scheduling issues (young working families) or a desire for greater anonymity. After careful consideration, we opted to interview both members of the couple together. We believed this would help us best understand the joint experiences of each couple, and ensure that both partners were aware of the information shared [26]. The qualitative researcher (TN) and two of the interviewers (ST, DJ) developed the interview guide based on a review of literature. However, given the paucity of literature documenting the experiences of PLWHIV seeking fertility treatment, we adopted a semi-structured format with open-ended questions to encourage participants to express personal experiences within each domain that were important to them.

Questions were asked following a semi-structured interview guide, addressing the following domains: 1) desire to have children, 2) worries concerning conception, 3) feelings regarding fertility, 4) impact on lifestyle, 5) support networks, 6) physician counselling on pregnancy planning received, 7) sources of information on pregnancy planning in the context of HIV, 8) opinion on the current available support, and 9) satisfaction with fertility planning experience. Within each domain, participants were asked for recommendations of ways their experiences could have been improved. The interviews lasted ninety minutes to two hours in duration and were audio-recorded.

### Data Analysis

The audio-recorded interviews were transcribed and inputted in NVivo as they were completed. Interviews went through a first stage of primary coding by the qualitative researcher wherein codes were developed. Coded interviews were then analysed by an iterative process of constant comparison to identify both recurring themes and the range of variation and nuance across participants' narratives. The qualitative analyses were then structured around themes identified as central to understanding participants' experiences, desires and opinions around pregnancy and themes that emerged from the data itself such as the level of support from various networks (family, medical) as well as factors identified (age, race, ethnicity, gender) that influenced pregnancy planning for these couples. Member checking was not employed in this research project. Research team members participated in the analysis and interpretation of the data. Saturation was achieved after 12 interviews when no new themes or experiences emerged from the data and enrolment was closed.

## Results

### Sample Demographics

All participants lived in Ontario and all but three were of Caucasian descent. The average age for female participants was 37 and 38 for male participants. The majority had some or completed college or university education. Of those who disclosed household income, all but one had household incomes of $45,000 or greater. A majority of women and just over half of the men were employed. Four men were on permanent disability, receiving a settlement package offered by the government related to the acquisition of HIV through blood products. Eight of the men were haemophiliacs who received HIV infected blood products, 3 identified as men who have sex with men, and one was from an endemic country. The couples had a total 9 biological children together (and 3 couples were pregnant at time of interview) utilising sperm washing. In addition, one couple conceived naturally and two used donor insemination. All men who utilised sperm washing services had undetectable (less than 50 copies/mL) viral loads at the time of insemination- only one had to change his cART to reduce his viral load from initial assessment to an undetectable level at insemination.

### Motivations for Pregnancy

#### The Lazarus Effect: Coming back to a “normal” life

From the start of the pandemic and discovery that HIV could be transmitted vertically and horizontally, couples living with HIV were discouraged from pursuing pregnancy and other ways of having children (adoption, sperm donor) given the low life-expectancy and risks of HIV transmission [3]. Over half of the male participants in our sample were haemophiliacs who had been diagnosed in the 1980s when life expectancy was low and treatment was not available. Couples put off their expectations to have children and devoted the time they thought they had left to preserving longevity. Respondents, notably those diagnosed early on in the pandemic, expressed a sense that with the development of cART, they had been brought back from near death (the Lazarus effect) and they now had a chance at a ‘normal’ life that included having their own biological children [27].

*My view of my future has change dramatically since I first got diagnosed and to where I am today. The doctor told me at the beginning, don't bother getting married, you're not going to last long. There's not really a great chance that you'll be here, even a year. So now, they refer to this disease as a chronic disease, and so I feel that, you know, with great medical help and amazing drugs that keep coming, I feel like I could have almost a normal life expectancy. So I have great hope.*

#### Exploring other options

Although cART had a dramatic impact on life expectancy and the prospect of raising a family became a possibility for couples living with HIV, fertility options were very limited, particularly in North America. With the exception of few clinics in the US working with PLWHIV, only a few options were available to PLWHIV such as adoption or sperm donor insemination [3]. However, even these options were not always available to the HIV community given the stigma associated with HIV and the perception that HIV-discordant couples should not have the same access to pregnancy, fertility and adoptions services [2]. Three of our study participants previously used donor sperm or adoption as a way to construct their family, accepting that the male partner would not be the biologic father.

*Murray had basically resigned himself to the fact that he would never have biological children. He would have children but that they would be by donor insemination.*

Two couples encountered resistance when searching for a clinic that was open to inseminating donor sperm into a woman whose partner was living with HIV. Two couples said they were treated differently by adoption agencies because of their HIV status – they were told they would only be able to adopt HIV-positive children or children with disabilities. Some couples were concerned that the required medical examinations would expose their HIV status and thereby disqualify them for adoption.

*Stephane: But yeah, we did think of adoption and we delayed for a long time inquiring about it because we figured that HIV and the stigma associated with it was a barrier. And finally we went to a little info session and we met a social worker there that encouraged us to apply. And it was about maybe four years ago Jasmine?*

*Jasmine: Yeah.*

*Stephane: We went through the process and we got approved – we got our home study approved and we were on the waiting list, but we never got anywhere.*

*Jasmine: Well that's not actually true. They did try to have us adopt a child that had severe FAS [fetal alcohol syndrome] and they felt that it would be a good suit for us because we have managed with so many other difficulties in our life.*

#### ‘I wanted my boy to look like me’ – Paternal and couple motivations

The implementation of cART had a significant impact on the health, well-being and life-expectancy on many of our couples. Conceiving children and raising a family became a possibility for couples living with HIV. Once those fertility desires could be realized, the motivation was not solely this new chance of life but also strong individual and couple desires to not only have children but also to bear their own biological children which speaks to social norms regarding family, relationships and gender. In addition, some men felt that it was their duty and responsibility to give their partners children and that because of their HIV status, they had failed this part of the relationship – this highlights the expectations and sense of responsibility placed upon men as partners/husbands and as fathers. The decision-making and discussion around pregnancy, family and HIV amongst the couples reveals the gendered assumptions and expectations (as well as intersection of race, class, age) that shapes and structures this motivation. It also demonstrates the social norm that heterosexual couples are expected to produce their own biological offspring and that any other means (donor sperm, adoption) are viewed as inferior options.

*I was always kind of worried that Shannon would resent me to some degree, because I can't father children for her. And, that was a worry as well so the fertility treatments were a Godsend.*

Some couples, did not want to have children unless both members of the couple were the biological parents, so they put off the idea of having a family given the lack of available fertility services. The motivations of the female partner played a significant role in the decision-making around how a child would be conceived as it potentially put them at risk for infection. On a social level, the women interviewed experienced stigma being in a relationship with a HIV-positive man as well as potentially producing a child with a partner with an ill parent.

*I kept pushing and pushing and pushing and I tried different things and different ways and a lot of it wasn't agreeing with Greg because I was discussing sperm donors and stuff like that and he would get upset but not really talk about it and I got the drift that his feeling was well, if the child can't be part mine then it can't be part yours either and let's try for just straight adoption.*

There was a sense, often expressed by both parents that the child should look like his father.

*We both knew we wanted babies. I as a Dad, a man, I wanted my boy to look like me. And as far as I'm concerned, that's why the natural stuff came up and the sperm washing came up.*

Also, given that many of the couples were not open about their HIV status, they worried that having children that didn't look like the father would precipitate questions as to the need for donor insemination– and this would force HIV disclosure and accompanying stigma.

*We haven't really talked to many people about it, because lots of questions come up, and people want to know why you are going to a fertility clinic? We don't really lie, we just say that we have fertility issues and that's why we are going to a fertility clinic, and kind of leave it at that.*

The desire and need to have a biological child was strong for several couples. In one case, the couple conceived naturally despite the concerns (mostly of the father) of HIV transmission. For several of our couples, the motivation of the female partner was a key factor in the fertility intentions and actions of the couple.

#### ‘Where is the baby?’ – External, family pressure

Outside the desire to have biological children, other factors contributed to the motivation to have a child. Many couples were not open about their HIV status to friends and family and the lack of children in the relationship presented a problem. Given their age and marital status, they felt it was expected that they should have children.

*Chuck: You know people saying, when are you going to have kids? And how do you answer that? And why don't you have kids and…?*

*Elise: Especially, I think, I get it a little bit on my side, more so, because they know that Chuck has a daughter. So they are kind of like, you've never been married, you've never had kids, like, what is wrong with you?*

By not having children, some couples worried that others would suspect HIV. This fear of disclosure was particularly relevant to the men with haemophilia who felt those around them suspected they were infected due to the public association of haemophilia with HIV via contaminated transfusions.

*Kirk: A lot of people don't even know my haemophilia status just because of the fact that along with haemophilia so many people got infected with HIV, so we even…*

*Hazel: We lie.*

*Kirk: We won't even tell people that (haemophilia), right. Not even your brother knows.*

Some couples, after HIV diagnosis, adopted a ‘childless’ lifestyle where work, travel, etc. made up for the lack of children without bringing into question the health status of the couple.

*I think family, certainly mine, knew the situation, knew it wasn't possible, and we acted, we carried our lives on it that if it wasn't ever–wasn't something we were trying to do, we were kind of the ultra-modern couple that didn't do any of that stuff, that had kids, you know. Or that's what we first did, that's what we portrayed anyway.*

### Access

At the time of the study, only two clinics in Ontario provided sperm washing services to couples living with HIV. In our sample, several of the participants' HCPs were not aware of sperm washing or only knew of clinics located in Europe or the US and hence cost was seen to be prohibitive.

For many, the option of travelling to the US or Europe to have the procedure done was too costly and not feasible. In 2001, a clinic in London, Ontario, the first in Canada, began offering sperm washing services to the HIV community. Respondents were surprised at the relative affordability of the procedure ($150–200 for sperm washing and IUI). For most couples in our study, this cost was minor compared to anticipated costs of seeking treatment in the US or Europe. Many respondents expected to pay thousands of dollars for the procedure and the assumed expense of the procedure had been a barrier for several couples.

*Jasmine: We had originally planned to pay thousands, and thousands, and thousands of dollars in fact, but we've always been so, we didn't know what in-vitro was going to cost us, but we were actually pleasantly surprised how that was, I think that was 150 dollars for one insemination. That's IUI treatment, and then the cost of my drugs only for one week out of my cycle, and the cost wasn't very much money. And so… and I think you just come to a point where you are so desperate for a child, it doesn't matter. You will find a way to get the money to get the baby, or try and have the baby.*

#### Accessible but stressful

In our study, once couples were aware of the fertility services available to them in Ontario, most were able to afford the services, with some having costs covered by various compensation packages. However, many couples found that distance to the clinic limited access to services, as each appointment required up to 8 hours for transit and time spent waiting at the clinic and receiving fertility services. Although they felt it worthwhile, respondents mentioned travel and time to be significant sources of stress, particularly for those who were not open about why they needed to go so far for fertility appointments.

*We were stressed out. It was a difficult time because we were working, and we were driving to London at like four o'clock in the morning and going and doing stuff and coming back for work. So it was a stressful time, but the act itself, when we were in there and what was being done,… like it was clinical and everything, it was still a special moment because it was the hope of something more out of the two of us.*

For most couples, both members were employed and required frequent and lengthy absences from work to attend fertility clinic appointments, making it particularly problematic for couples who were not open about their HIV status. Asking for time off to visit a fertility clinic raised many questions. Why did the couple need to use fertility services? Why did the couple need to travel so far to use that particular clinic's services where there are so many in their own city?

Other couples were open with their employers about why they had to travel to receive services and most couples found them to be supportive.

*Myriam: By that time when we did the examination I was transferred to another position in the business so they understood – and I only discussed this with my manager. She knows of the status – but I went to tell them I'm going to try a fertility clinic so I will – I needed some days – and they were very understanding.*

*Interviewer: They knew about the HIV status as well?*

*Myriam: Only the manager.*

*Interviewer; So she knew why you had to go to London as opposed to coming here.*

*Myriam: Exactly. Because they know there are hundreds of fertility clinics in Toronto, why London?*

For the majority of couples, the concern about the risk of infecting the woman and possibly fetus was too great to attempt natural conception. However, one couple felt comfortable taking a ‘calculated risk’ of infection by having unprotected sex, as the male partner had undetectable serum viral loads. This couple conceived naturally and no HIV transmission resulted. However, when they were planning for their second child, they chose to use sperm washing services rather than take another ‘calculated risk’ feeling the risk was no longer acceptable to them. Access to fertility services such as sperm washing gave couples an option to reduce their risk of horizontal transmission.

### Knowledge

Participants identified their own research (through the Internet and medical journals), media, communication with other couples in similar situations, and a few HCPs (particularly a knowledgeable haemophilia nurse who initiated discussions with participants about their fertility desires) as initial sources for information about fertility services. Not every HCP or fertility clinic, however, was knowledgeable about sperm washing, or receptive to hear information presented by their patients.

*Peter: Bethany and I did a lot of research ourselves, … and so, we went there with a lot of questions about the procedure itself, … when we went to the doctor in Hamilton, it was depressing, because they didn't even give us the time of day, they flat out told us that there was no hope at all that we would ever have a baby. And I think that, that was the most frustrating time for us. Because we knew that there was a procedure out there*

*Bethany: And nothing was, none of our questions were being answered, and the doctor actually got up and left. And by the time, we couldn't even had the chance to digest the fact that we've just been told that we were never going to have a baby. This lady walked in and started talking to us about sperm donor and adoption and it's so, a slap in the face because we didn't even have the chance to stop and address the specific goal, before the next person came in and telling us about something that we didn't want down our road.*

Interviews in our study revealed that for the most part, couples had to gather their own information because it was not readily available from the medical or HIV communities. A few couples heard news reports that featured a sperm washing clinic in Italy, encouraging them to further investigate their options. The advent of the Internet proved to be useful as participants familiar with the keywords, ‘sperm washing’ or ‘HIV discordant,’ could search for information. Many had heard rumours that the procedure existed but were unsure as to where to locate reliable and accurate information.

Participants' desire for information ranged greatly- some wanted a basic informational pamphlet, whereas others wanted more detail, such as access to scholarly articles and research. Recommendations from our couples included having information provided in a variety of languages, and information targeted to family, friends and others involved to help them understand the safety of the procedure. Participants also made it quite clear that relevant up-to-date information needed to be conveyed to HCPs, nurses, doctors, and fertility clinics. Many respondents found out about the one available fertility clinic in Ontario through one nurse who worked at their haemophiliac clinic.

*Josee (a nurse at the clinic), this woman…went above and beyond the call of duty. And I think she did what she could for us, I don't think she had any more time, but it would have been nice if someone would have said “Hey!” You know, Ronald, there is a young group of men who are, who are living with this, and are having full lives, it would have been nice if someone was able to sort of bounce off Josee's enthusiasm and say this is something that is affecting our patients we need to learn more about this, and we need to provide a support system for them or we need to find out why these people have to go to London to get this when Toronto, how many major hospitals are there in this city. And we were going to a little, small private fertility clinic.*

Some respondents felt that they were the ones educating the HCPs and reported a lack of knowledge about the procedure (safety, availability, cost). In the end, most HCPs were supportive of couples once they had been educated about the sperm washing procedure, its safety and availability within Ontario.

*Kyle: Well I guess considering what was available, once we were connected with the right people it went pretty well, it just would have been nice if it wasn't such a struggle to find someone who was willing to be on board. I mean, my doctor was willing to help out, it would have been nice to have someone who already had the knowledge and…*

*Melanie:… even if there was literature for, even for a physician to read I think they would be more knowledgeable and therefore be more open minded to what we were doing.*

For many couples, only their HCPs knew of their HIV status. Accordingly, participants felt that HCPs should ask their patients if they have ever thought of having children and if they knew about safer options to facilitate this process. Participants also wanted information pamphlets available at other locales, such as their various doctors' offices and AIDS service organizations (ASOs). Only two couples in our study were directly asked by their physician or nurse if they wanted to have children. One couple noted that while there was some HIV and pregnancy information available via ASOs, it was only targeted to women living with HIV and there was a need for information that included men living with HIV and their partners.

### Support

Couples in our study reported receiving support from a variety of sources, but the level of support depended greatly on the couples' level of HIV disclosure. Respondents identified two main sources of support: HCPs and family and/or friends. Couples also mentioned the possibility of acting as support for other couples in similar situations.

#### Medical Field

Similar to varying levels of knowledge, levels of support from HCPs also varied vastly. Couples reported that the type and level of support depended on their relationship with the HCP and on the personality and role of the HCP. Many reported that specialists (HIV, haemophilia) focused on specific clinical issues and were not concerned with the other components of their well-being, such as family planning.

Couples reported mixed reactions and support from their family physicians – from outright rejection of the possibility of pregnancy to full support, even if the physicians knew little about the procedure. Couples often took on the role of educator – providing their family physician with medical articles, etc. However, this education only really occurred with physicians who were receptive to discussing the possibility of pregnancy in the context of HIV. Several couples changed family physicians to find a more supportive environment.

Couples had mixed experiences with fertility clinics as well. One couple received sperm washing services considered an ‘experimental’ procedure from a physician in the 1990s but after an unsuccessful attempt, the clinic felt it could not ethically support further attempts. Another couple was referred by a HIV physician to a fertility clinic and was refused treatment based on their HIV status. On the other hand, couples reported their experience at the facility that pioneered access to the procedure in Ontario to be very supportive – refreshingly so for those couples who experienced rejection and discrimination at other locales.

#### Families, friends

Couples relied on the support of family and friends who were aware of their HIV status. In situations where HIV status was not disclosed, couples still received support, but this often required more ‘work’ on the part of the couples – in justifying the need to travel for fertility services, particularly, when many clinics existed nearby. In our study, generally, the male's family was aware of his HIV status and in most instances, the female's immediate family was also aware. The support of family proved to be important in couples' sperm washing experiences.

*My mom and dad knew how much we wanted children and when Nathaniel couldn't take me, my dad didn't want me driving by myself so he would come here, and pick me up at 4 o'clock in the morning and he would, I would drive down, and he would sit with me in the waiting room and keep me company, and then he would drive me back, well he would drive back so that I could sleep, like just we, our parents really were wonderful and supportive, I mean we couldn't have asked for better parents.*

However, not all family and friends were perceived to be supportive and as a result couples were selective in whom they disclosed HIV and fertility information to. One couple, anticipating a negative reaction, misled the woman's family as to the reason they were travelling all the way to London for fertility treatment. They only revealed the true reason, his HIV status, after she had become pregnant.

*Interviewer: So at this point people started to know that you were HIV positive*

*Marjorie: Yes, we told my mom and then I guess…*

*Interviewer: Were they supportive? Was your family?*

*Marjorie: Oh, my parents flipped off the roof because I was pregnant.*

*Interviewer: Right.*

*Marjorie: And they didn't know any of this. They thought I was going to London because I…*

*Harvey: To visit the queen.*

*Marjorie: No, because I had found a great fertility doctor and…*

*Interviewer: So they thought you were going for fertility because they thought you couldn't get pregnant?*

*Marjorie: They thought Harvey had a low sperm count.*

*Harvey: Is that what you told them?*

*Marjorie: Yeah. And then…did I ever tell you that? Well I had to explain it somehow because it's not my…it's not my secret to tell.*

Again, the reaction of familial and friendship networks ranged from overwhelming support to outright rejection. Knowledge of the procedure and its relative safety informed peoples' understanding of the process and also increased the support from friends and family members. Participants identified support from all parties involved in the pregnancy process as important - from the HCPs involved with testing and monitoring the pregnancy to the support of families and friends who provided care and guidance during and after the pregnancy. The varying levels of support often depended greatly on their level of HIV disclosure. A few couples did it all on their own – dealt with the stress of travel, high cost, failed pregnancies – because they were not comfortable disclosing their HIV status. Other couples, often politically and socially involved with various haemophilia associations, gained the support of those around them. The couples who were open about their HIV status were also more likely to act as mentors and sources of information for other couples in their situation.

### Stigma and Secrecy

The ongoing stigma surrounding HIV, and more specifically in this study, around HIV-discordant couples, was one of the key issues identified. Stigma had a profound affect on all components of the decision-making that surrounds the pregnancy process – motivation, knowledge, access and support. Men who were infected with HIV through blood products – often perceived as ‘innocent victims’ – experienced stigma differently than those infected through sexual contact or injection drug use and this could have resulted in increased support from some HCPs and family members.

However, there is still a larger stigma that surrounds HIV and pregnancy given the potential for horizontal and vertical transmission and the continuing social stigma of HIV, illness, responsibility and mortality. This stigma had an affect on the types of support couples received from their social and employment networks. Couples were concerned that their children would be stigmatised if the father's HIV status were known irrespective of his HIV risk factor. There was great variability amongst our sample regarding opinions about whether the children should be informed about the father's status. Some couples were or planned to be open with their children about their HIV status and others kept it a well-guarded secret.

*Myriam: I think also, there is the issue of being a very religious family and having a very like, very important relationship at our church that there are, the questions that everybody would then want to be answered, like people who think they deserve to know how this happened, which then involves basically embarrassment or whatever.*

*Hugo: I feel that since there is, especially a long time ago it was a huge stigma attached with being HIV positive. I was very concerned for, you know, that I might be judged or I might be persecuted basically.*

Given the negative reaction and lack of knowledge demonstrated by some medical professionals, some respondents were seriously concerned about the reaction by the ‘general public’ and thus chose not to disclose their status to anyone outside the relationship. Couples not open about their HIV status were concerned about the perceptions and judgements if others knew about their HIV status in the context of their fertility intentions and actions. This stigma was experienced by both couples who already had children and those without children. Stigma was layered throughout the experiences of participants from the stigma of using fertility services (viewed as unnatural by some religions), the stigma of being a childless couple, the stigma of being HIV positive, the stigma of potentially having HIV positive children and the stigma of not being about to adequately care for children. The perceived, enacted and internalised stigma was experienced by all couples on some level and there was some debate amongst couples about if and when the father's status should be disclosed to the children.

## Discussion

As in other studies, we found that the desire to have children has always existed with PLWHIV, but what has changed is the fertility possibilities and expectations for this community [3], [10]. The diagnosis of HIV during the 1980s or 1990s did not dampen the desire of our participants to have children, but did quell the expectation for biological offspring. The desires, motivations and decision-making to have children are situated within complex social and historical contexts (gender roles, family and social expectations, the evolution of HIV in society). Due to the stigmatized nature of HIV, most couples limited the disclosure of his HIV status but in doing so experienced the social norms and expectations of couples to produce children.

Studies have found that the desire and intention to have biological children, is so strong that couples accept the risk of infection [9]. The Swiss statement in 2008 suggested that it was safe for HIV-discordant couples on antiretroviral therapy with undetectable viral loads and no concomitant sexual transmitted infections to conceive, but there has been much debate in the HIV medical community about this assertion given the fertility options now available to further reduce risk of transmission [28]. Due to the availability, albeit limited, of sperm washing services, HIV-discordant couples do not have to put themselves or their children at risk for HIV transmission and could realise their long held desires to safely have biological children. Limiting access to these services denies PLWHIV and partners their reproductive rights and forces some to risk HIV transmission through natural conception [3]. If PLWHIV were aware of the availability of fertility services, the relative ease, potential for low cost, and safety of the procedure, many may have chosen sperm washing over natural conception. More clinics need to offer fertility services to the HIV community to support the reproductive rights of PLWHIV.

Connected to the issue of access, our participants identified knowledge and information on the procedure as extremely important. Given that historically, PLWHIV were discouraged from becoming pregnant [3], up-to-date information on the safety of the procedures needs to be transmitted more effectively to all stakeholders. Several groups have now called for fair access to fertility procedures for PLWHIV [29]–[31] but service providers have been slow to respond [12]. Policy should be based on current medical evidence rather than on outdated understandings of HIV. Information about services (procedures available, safety) and access (cost, location) in Canada is poorly communicated to the HIV and medical communities. HCPs need to have a better understanding of the current evidence on sperm washing and other fertility services for PLWHIV. Encouragingly, in Canada, there has been the recent development of the National HIV Pregnancy Planning Guidelines by an interdisciplinary collaboration to provide evidence-based guidelines to assist Canadian PLWHIV and their HCPs [32]. More importantly, HCPs (physicians, nurses, social workers, etc.) need to raise the simple question to patients – ‘are you interested in having children?’ – because many PLWHIV don't know they can safely have their own children. The dynamics of heterosexual couples and the impact of gender roles and social norms in fertility decision-making need to be addressed by HCPs and service providers [33].

Outside of the medical community, there needs to be much more education about the pregnancy possibilities for men and women living with HIV. Information sessions have been arranged in the HIV community but often this information is targeted only to women living with HIV. One couple attended such a community meeting and had to educate the meeting leader on sperm washing. With more education and information, HIV-discordant couples can pursue pregnancy safely in a supportive environment and make informed choices.

Educating the general public will help to lessen the stigma of PLWHIV having children. Interestingly, a majority of male participants of this study were infected with HIV through contaminated blood products. We do not know if this represents a recruitment bias, or if a population that might be viewed as ‘innocent victims’ had increased access to services. With more education, support can be expanded to extend to PLWHIV, their family and friends, social service agencies, the medical community and ASOs. Stigma and the resulting secrecy add difficulty to an already stressful fertility process. Support for decisions around pregnancy has been shown to be important for couples generally but for HIV-discordant couples that require fertility services, support is even more crucial [30], [31].

Our study is the first to document the experiences of HIV-discordant couples in Ontario pursuing fertility services to reduce the risk of HIV transmission. However, we acknowledge there were some limitations that may limit the applicability of our findings to the general HIV-discordant population. We did not pilot the interview guide nor did we employ member checking. Our sample size was small and recruitment was done only via the HIV HCPs and the fertility clinic. Thus, we may have missed Ontario couples who left the country for the procedure. In retrospect, we could have expanded our recruitment strategies to include flyers, posters, snowball referral etc., and engaged ASOs and haemophiliac clinics in recruitment. Finally, the generalizability of results is a limitation as our study population consisted mainly of Caucasians who were highly educated and employed and is not representative of the general HIV population in Ontario. An extensive discussion and analysis of the research challenges encountered in our study of HIV-discordant couples seeking fertility treatment can be found in Tecimer et. al [26].

In our study, the key issues for the HIV-discordant couples pursuing fertility treatments to reduce HIV transmission risk included motivation to have children, access to services, lack of knowledge about available procedures, lack of support and stigma. By documenting the experience of HIV-discordant couples that have pursued sperm washing in Ontario, this study allows stakeholders such healthcare and social service providers, ASOs, and the HIV community to become aware of the gaps in knowledge and access so that more options can be facilitated for PLWHIV. The current body of evidence supports sperm washing as a safe procedure with no documented cases of vertical or horizontal HIV transmission. PLWHIV, like the general population, desire to have children and raise families and should be fully supported in this decision. Limiting access to sperm washing procedures is denying PLWHIV of their fundamental reproductive rights.

This study has important implications for HIV- discordant couples that HCPs and policy makers should consider. Health care providers must consider adding a discussion about contraception, pregnancy planning, and healthy pre-conception into routine HIV care. Doing so will support safer pregnancies, maximize the health of couples by reducing horizontal transmission risk and protect future children by reducing vertical transmission risk. Canada is in the process of developing national guidelines on pregnancy planning as well as provincial and national HIV Fertility Programs [18], [30], [32]. We hope that our research and ongoing projects assist HIV-positive individuals, policy makers and HCPs globally to develop programmes for safer, supportive pregnancy and family planning for individuals and communities affected by HIV.

## References

[1] Lima VD, Hogg RS, Harrigan PR, Moore D, Yip B (2007). Continued improvement in survival among HIV-infected individuals with newer forms of highly active antiretroviral therapy.. AIDS.

[2] Klein J, Pena JE, Thornton MH, Sauer MV (2003). Understanding the motivations, concerns, and desires of human immunodeficiency virus 1-serodiscordant couples wishing to have children through assisted reproduction.. Obstet Gynecol.

[3] Gruskin S, Ferguson L, O'Malley J (2007). Ensuring sexual and reproductive health for people living with HIV: an overview of key human rights, policy and health systems issues.. Reprod Health Matters.

[4] Cooper ER, Charurat M, Mofenson L, Hanson IC, Pitt J (2002). Combination antiretroviral strategies for the treatment of pregnant HIV-1-infected women and prevention of perinatal HIV-1 transmission.. J Acquir Immune Defic Syndr.

[5] Loutfy MR, Hart TA, Mohammed SS, Su D, Ralph ED (2009). Fertility Desires and Intentions of HIV-Positive Women of Reproductive Age in Ontario, Canada: A Cross-Sectional Study.. PLoS ONE.

[6] Panozzo L, Battegay M, Friedl A, Vernazza PL, Swiss HIV Cohort Study (2003). High risk behaviour and fertility desires among heterosexual HIV-positive patients with a serodiscordant partner–two challenging issues.. Swiss Med Wkly.

[7] Chen J, Phillips K, Kanouse D, Collins R, Miu A (2001). Fertility Desires and Intentions of HIV-Positive Men and Women.. Fam Plann Perspect.

[8] Public Health Agency of Canada (2009). HIV and AIDS in Canada..

[9] Sherr L, Barry N (2004). Fatherhood and HIV-positive heterosexual men.. HIV Med.

[10] Bujan L, Hollander L, Coudert M, Gilling-Smith C, Vucetich A (2007). Safety and efficacy of sperm washing in HIV-1-serodiscordant couples where the male is infected: results from the European CREAThE network.. AIDS.

[11] Semprini A, Lei-Setti P, Bozzo M, Ravizza M, Taglioretti A (1992). Insemination of HIV-negative women with processed semen of HIV-positive partners.. Lancet.

[12] Yudin MH, Shapiro HM, Loutfy MR (2010). Access to infertility services in Canada for HIV-positive individuals and couples: a cross-sectional study.. Reproductive Health.

[13] Sunderam S, Hollander L, Macaluso M, Vucetich A, Jamieson DJ (2008). Safe Conception for HIV Discordant Couples through Sperm-Washing: Experience and Perceptions of Patients in Milan, Italy.. Reprod Health Matters.

[14] Paiva V, Filipe EV, Santos N, Lima TN, Segurado A (2003). The right to love: the desire for parenthood among men living with HIV.. Reprod Health Matters.

[15] Cooper D, Harries J, Myer L, Orner P, Bracken H (2007). “Life is still going on”: Reproductive intentions among HIV-positive women and men in South Africa.. Soc Sci Med.

[16] Oosterhoff P, Anh NT, Hanh NT, Yen PN, Wright P (2008). Holding the line: family responses to pregnancy and the desire for a child in the context of HIV in Vietnam.. Cult Health Sex.

[17] Yudin MH, Money D, Cheung M, Loutfy MR (2011). Canadian physician attitudes regarding pregnancy, fertility care, and assisted reproductive technologies for HIV-positive individuals or couples.. Abstract Program at 20th Canadian Association of HIV Research Conference.

[18] Margolese S, Loutfy MR, Huyhn L, Conway T, Maxwell J (2010). Improving access to pregnancy planning and reproductive options for people living with HIV through evidence based policy development and advocacy.. Abstract Program at the XVIII International AIDS Conference.

[19] Nattabi B, Li J, Thompson SC, Orach CG, Earnest J (2009). A Systematic Review of Factors Influencing Fertility Desires and Intentions Among People Living with HIV/AIDS: Implications for Policy and Service Delivery.. AIDS Behav.

[20] Kanniappan S, Jeyapaul MJ, Kalyanwala S (2008). Desire for motherhood: exploring HIV-positive women's desires, intentions and decision-making in attaining motherhood.. AIDS Care.

[21] Ko NY, Muecke M (2005). To reproduce or not: HIV-concordant couples make a critical decision during pregnancy.. J Midwifery Wome Heal.

[22] Siegel K, Schrimshaw EW (2001). Reasons and justifications for considering pregnancy among women living with HIV/AIDS.. Psycho Women Quart.

[23] Myer L, Morroni C, Rebe K (2007). Prevalence and Determinants of Fertility Intentions of HIV-Infected Women and Men Receiving Antiretroviral Therapy in South Africa.. AIDS Patient Care and ST.

[24] Lurie M, Pronyk P, de Moor E, Heyer A, de Bruyn G (2008). Sexual behavior and reproductive health among HIV-infected patients in urban and rural South Africa.. J Acquir Immune Defic Syndr.

[25] Myer L, Morroni C, Cooper D (2006). Community attitudes towards sexual activity and childbearing by HIV-positive people in South Africa.. AIDS Care.

[26] Tecimer SN, Jaworsky D, Newmeyer T, Chihrin S, Gough K (2011). Learning from Interviews with HIV-Discordant Couples (Male Positive, Female Negative): The Challenges and Successes.. Open AIDS.

[27] Scott S, Constantine L (Jul–Aug). The Lazarus syndrome: a second chance for life with HIV infection.. J Am Pharm Assoc.

[28] Vernazza P, Hirschel B, Bernasconi E, Flepp M (2008). HIV-positive individuals without additional sexually transmitted diseases (STD) and on effective anti-retroviral therapy are sexually non-infectious.. Bull des Medecins Suisses.

[29] The Ethics Committee of the American Society for Reproductive Medicine (2010). Human immunodeficiency virus and infertility treatment.. Fertil Steril.

[30] Yudin MH, Loutfy MR (2011). Advocating for Assistance with Pregnancy Planning in HIV-Positive Individuals and Couples: An Idea Whose Time Has Come.. J Obstet Gynaecol Can.

[31] Gruskin S, Firestone R, MacCarthy S, Ferguson L (2008). HIV and Pregnancy Intentions: Do Services Adequately Respond to Women's Needs?. Am J Public Health.

[32] Margolese S, Carvalhal A, Chu S, Gysler M, Hamilton S (2011). National HIV Pregnancy Planning Guidelines Developed to Assist People Living with HIV in Canada with Their Conception Planning and Fertility Needs.. Abstract Program of the 1st International Workshop on HIV & Women, from Adolescence through Menopause.

[33] Vandevanter N, Stuart Thacker A, Bass G, Arnold M (n.d.). Heterosexual couples confronting the challenges of HIV infection.. AIDS Care.

